# Preoperative external beam radiotherapy and reduced dose brachytherapy for carcinoma of the cervix: survival and pathological response

**DOI:** 10.1186/1748-717X-2-9

**Published:** 2007-02-22

**Authors:** Alexandre A Jacinto, Marcus S Castilho, Paulo ERS Novaes, Pablo R Novick, Gustavo A Viani, João V Salvajoli, Robson Ferrigno, Antonio Cássio A Pellizzon, Stella SS Lima, Maria AC Maia, Ricardo C Fogaroli

**Affiliations:** 1 Department of Radiation Oncology, Hospital do Cancer A C Camargo, São Paulo, Brazil; 2 Department of Gynecology Oncology, Hospital do Cancer A C Camargo, São Paulo, Brazil

## Abstract

**Purpose:**

To evaluate the pathologic response of cervical carcinoma to external beam radiotherapy (EBRT) and high dose rate brachytherapy (HDRB) and outcome.

**Materials and methods:**

Between 1992 and 2001, 67 patients with cervical carcinoma were submitted to preoperative radiotherapy. Sixty-five patients were stage IIb. Preoperative treatment included 45 Gy EBRT and 12 Gy HDRB. Patients were submitted to surgery after a mean time of 82 days. Lymphadenectomy was performed in 81% of patients. Eleven patients with residual cervix residual disease on pathological specimen were submitted to 2 additional insertions of HDRB.

**Results:**

median follow up was 72 months. Five-year cause specific survival was 75%, overall survival 65%, local control 95%. Complete pelvic pathological response was seen in 40%. Surgery performed later than 80 days was associated with pathological response. Pelvic nodal involvement was found in 12%. Complete pelvic pathological response and negative lymphnodes were associated with better outcome (p = .03 and p = .005). Late grade 3 and 4 urinary and intestinal adverse effects were seen in 12 and 2% of patients.

**Conclusion:**

Time allowed between RT and surgery correlated with pathological response. Pelvic pathological response was associated with improved outcome. Postoperative additional HDRB did not improve therapeutic results. Treatment was well tolerated.

## Background

Radiotherapy (RT), surgery (S), or the combination of both treatments with preoperative radiotherapy following surgery (RT→S) have all been shown to be effective local-regional treatments [1-8] for patients with FIGO stages IB1, IB2, IIA and IIB (with <1/3 proximal parametrial invasion) cervix carcinoma [9,10]. Recent randomized trials have demonstrated that the addition of chemotherapy (CT) to RT improves treatment results [11,12]. The choice of the best local-regional approach remains controversial. Early retrospective reviews showed better results for patients treated with hysterectomy following radiotherapy for bulky cervical carcinoma [13,14]. O'Quim and cols published special recommendations for hysterectomy following RT for bulky endocervical carcinoma [15], but more recent randomized and retrospective studies have failed to demonstrate better local control or survival with such combined modality [3,16-18] and therefore RT→S remains controversial.

Several factors have been associated with prognosis for patients with cervical cancer treated with RT followed by surgery: performance status, age, tumor size, FIGO stage, residual tumor, histology, and nodal status [4,6,17,19]. There is no consensus on whether or not the presence of residual tumor on hysterectomy specimens is related to better survival and local control [4,6,17,19-21]. Few studies have evaluated the role of external beam radiation therapy and brachytherapy with high dose rate (HDRB) as a preoperative modality.

We performed a retrospective study to analyze the pathologic response and to relate it to survival in patients with early stage cervical carcinoma (most initial IIB) submitted to EBRT and HDRB following hysterectomy.

## Materials and methods

### Patients

from December 1992 to December 2001, 67 patients with invasive cervical cancer were submitted in a single institution to hysterectomy following preoperative radiotherapy with external beam irradiation and high dose rate brachytherapy. Chemotherapy was not administered to any of them. Median age was 46 years (range 22–72). Squamous cell carcinoma was the histological type in 56 patients (84%); adenocarcinoma in 9 (13%); and 2 patients (3%) had other histologies. Clinical staging of the tumor was defined after clinical history and physical examination performed at least by one gynecology oncologist surgeon and one radiation oncologist. According to the 1995 FIGO staging system 65 patients (97%) were "early" IIB (less than 1/3 proximal parametrial involvement), 1 (1.5%) was IIA and 1 (1.5%) was IB "bulky". All patients were submitted to cistoscopy, rectosigmoidoscopy, routine blood count, and biochemical profile and chest radiography. Abdominal-pelvic tomography was not routinely used until 1996, when it was incorporated to our staging routine for all patients. Patients' characteristics are shown in Table 1.

**Table 1:** Patient and treatment characteristics.

	Median	Range
Age	46	22 – 72
EBRT – Gy	45	29 – 45
HDRB – Gy	12	6 – 15
Radiotherapy duration – days	42	27 – 108
Delay to surgery – days	82	45 – 182

	Absolute number	%

Histological type		
Squamous cell carcinoma	56	84%
Adenocarcinoma	9	13%
Other	2	3
FIGO – Clinical stage		
IB2	1	1.5%
IIA	1	1.5%
IIB	65	97%
Pelvic lymphadenectomy	54	81%

### Radiation therapy

all patients received preoperative treatment with EBRT and reduced dose HDRB. Treatment with EBRT was delivered with 4 or 6 mV linear accelerators. Patients were treated in prone position with 45 Gy in a four-field "box'' technique to the whole pelvis. All fields were treated daily. Fractionation was 1.8 Gy per day five times per week. Median dose with EBRT was 45 Gy (range 29–45 Gy) and mean dose was 44.5 Gy. None of the patients received parametrial boost.

After the second week of pelvic irradiation all patients were submitted to a physical examination in order to evaluate the anatomical and geometrical conditions for brachytherapy, and whenever possible, high dose rate brachytherapy (HDRB) was started during EBRT. Intracavitary treatment (HDRB) was delivered with Fletcher after-loading applicators with an Iridium-192 source (IR-192) with a nominal activity of 10 Ci. Proposed dose to point A was delivered in two weekly insertions of 6 Gy. The median dose of brachytherapy to point A was 12 Gy (range 6–15 Gy) and the mean point A dose was 11.8 Gy.

According to the beliefs of the assistant physician, 11 patients with residual tumor on cervix and no positive margin on surgical specimens were submitted to postoperative vaginal vault HDRB with 12 Gy (2 fractions of 6 Gy) prescribed on the vaginal surface. Two other patients who presented cervical complete pathological response were also submitted to vaginal vault HDRB. The median time to complete both EBRT and HDRB was 42 days (range 27–108), and the mean time was 45 days.

### Surgery

The surgical procedure was carried out in a median time of 82 days (45 – 182) after the preoperative RT course (including the preoperative HDRB insertions). The procedure consisted of radical hysterectomy plus bilateral salpingo-oophorectomy – Piver II type. Fifty-four patients (81%) underwent selective pelvic lymph node dissection.

### Pathologic examination

Pathologic response was evaluated in the surgical specimens according the presence of residual tumor on the cervix, paracervical tissues and pelvic lymph nodes. Complete pathologic response (CPR) was defined as total absence of residual disease.

### Analysis of recurrent sites

Treatment failure was classified as local recurrence when it ocurred in cervix, paracervical tissues or vaginal vault. Whereas, local-regional recurrence when it occurred inside the pelvis. Distant metastasis was defined as any recurrence outside the pelvis.

### Statistical analysis

The chi-square test was performed to evaluate significance of variables. Kaplan-Meier test was used to calculate overall and specific survival. Univariate analysis was assessed using the log-rank-test.

### Analysis of complications

Complications were recorded for bladder, ureter, small bowel, and rectum. All acute and late complications were scored according to the Radiation Therapy Oncology Group (RTOG) scale.

## Results

**Figure 1:**
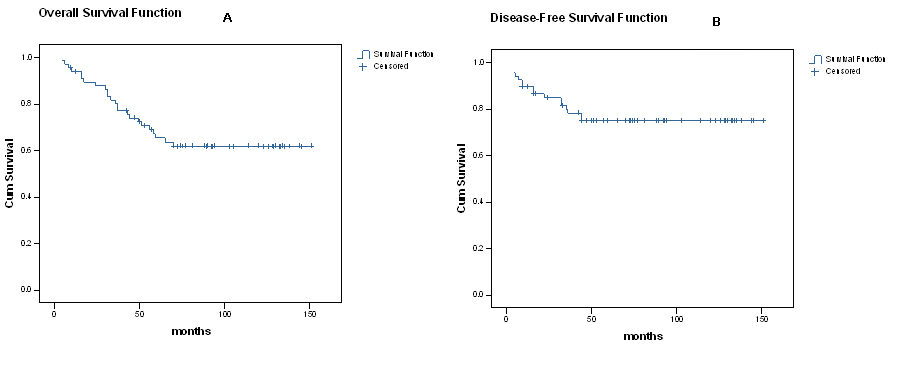
(a) Overall survival (OS) in 67 cervix cancer patients submitted to preoperative radiotherapy. (b) Disease free survival (DFS) of 67 patients submitted to preoperative radiotherapy.

Median follow-up time was 72 months (range 4 – 151). Two patients (3%) were lost to follow-up. At the end of this data collection, 41 patients (61%) were alive, of whom 39 had no evidence of disease. Sixteen patients (23%) died of cancer and 8 patients (12%) died of other causes. Five-year overall survival (OS) was 65%, and 5-year cause-specific survival (CSS) was 75% (Fig. 1a).

Local-regional recurrence occurred in 7 patients (10% – 3 local and 4 regional) and distant metastasis developed in 15 patients (22%). Five-year disease free survival (DFS), Local control, local-regional control and distant control were 75%, 95%, 90% and 79% (Fig. 1b).

Twenty-seven patients (40%) achieved pelvic complete pathological response (pCPR) – no residual tumor on any pathological specimen (cervix, parametrium and lymph nodes, if available). Cervical complete pathological response (cCPR) was found in 29 patients (43%). Parametrial CPR was achieved in all 65 patients with clinical parametrial involvement.

**Figure 2:**
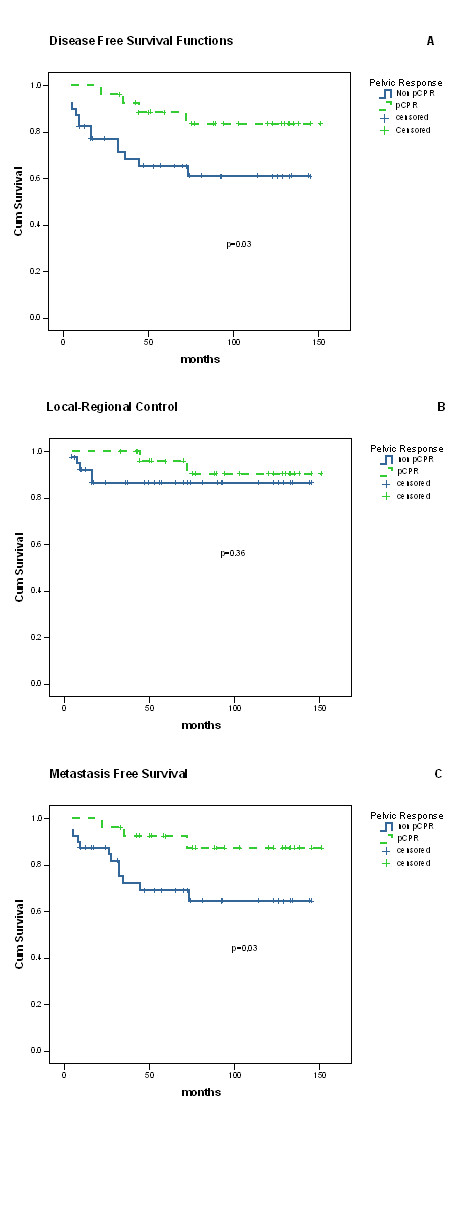
Survival in cervix cancer patients submitted to preoperative radiotherapy according to pathological pelvic response. (a) Disease free survival. (b) Local-regional control. (c) Metastasis tree survival. (pCPR: pelvic complete pathological response)

Five-year DFS was higher for patients who achieved pCPR (88% vs. 65%, p = 0.03). Also there was an advantage in 5-year distant control (92% vs. 69%, p = 0.03), but no significant statistic difference in 5-year local-regional control (96% vs. 86%, p = 0.3) (Fig. 2). Five-year overall and cause-specific survival were better for patients who achieved pCPR (72% vs. 54%, p = 0.06; and 86% vs. 63% p = 0.02).

For 29 patients with cCPR no recurrences were seen while for 38 patients with residual cervical tumors 3 recurrences occurred. However, these numbers did not reach significant level (p = 0.2). The 5-year OS, DFS, and CSS were 67% vs. 57% (p = 0.25), 82% vs. 69% (p = 0.19), and 85% vs. 67%, (p = 0.15).

For 11 patients with residual cervical tumors submitted postoperatively to vaginal vault HDRB there was one failure while for 27 patients with residual cervical cancer not submitted to postoperative HDRB there were 2 failures (10% vs. 7%; p = 0.97).

For the 54 patients submitted to lymphadenectomy (81% of the cohort) the median and mean number of lymph nodes dissected were 8 and 10 nodes respectively. Positive lymph node involvement (N+) was found in 8 patients (15%). Of 22 cCPR patients there were 2 N+ while among 35 patients with residual disease on the cervix there were 6 N+ (10 vs. 17%, p = 0.46). Lymph node involvement was a strong predictor of prognosis. Five-year OS for N+ and N- patients was 37% vs. 71% (p = 0.01), and 5-year CSS for N+ and N- patients was 46% vs. 78% (p = 0.01). Also, the 5-year DFS (80% vs. 47%, p = 0.005), 5-year metastasis free survival (84% vs. 47%; p = 0.0008) was worse for N+ patients, but the postoperative N stage had no impact on local regional control (93% vs. 87%; p = 0.57).

Median duration of radiotherapy was 42 days (range 27–108), and there was no significant statistic correlation between delay of irradiation and pathologic response on prognosis.

Patients underwent surgery after a median interval after radiotherapy of 82 days (range 45–182). When surgery was performed earlier than 80 days there were significantly less pCPR (22% vs. 57%; p = 0.003), and cCPR (28% vs. 57%; p = 0.017).

Age and histological type were not associated with prognosis or with better pathological response. (p = 0.3 and 0.14 respectively).

According to the RTOG morbidity scale there were 12% grade 3 or 4 late genitourinary and 4.5% late gastrointestinal sequelae. Table 2 shows the crude incidence of gastrointestinal and geniturinary complications.

**Table 2:** Crude incidence of toxicity according to the RTOG criteria.

Grade		0	1	2	3	4
Genitourinary tract	Acute	57(85%)	5(7.5%)	5(75%)	0	0
	Late	53(79%)	2(3%)	4(6%)	4(6%)	4(6%)
Gastrointestinal Tract	Acute	37(55%)	18(27%)	12(18%)	0	0
	Late	57(85%)	5(7.5%)	2(3%)	2(3%)	1(1.5%)

## Discussion

In the late 60's Durrance and cols published their analysis of cervical cancer central recurrences from a retrospective study conducted in the MDACC. They showed that after radical radiotherapy the incidence of central recurrences was higher in patients with bulky or barrel-shaped disease, and that local control could be improved with post irradiation histerectomy. However, they have included patients with extensive parametrial disease [14]. In the mid 70's Rutledge and cols published another study, from the same institution. This time excluding patients with massive tumors, and confirmed the concept that the addition of post irradiation surgery to bulky disease patients improved results in local control [13]. During this period, in Europe, Pilleron and cols used this modality of treatment published in the Institute Curie and showed worse local regional and distant control in patients with residual tumor after preoperative brachytherapy [22].

Based on these studies and in other smaller reports, Nelson and O'Quin introduced guidelines for hysterectomy after irradiation [15,23]. Several institutions around the world then adopted pre-operative irradiation as the standard treatment of bulky uterine cervical cancer and new conflicting data began to appear.

In the late 80's the first drawback came when Perez and cols published a prospective randomized trial and described comparable results with either surgery following radiotherapy or radiotherapy alone [24]. Perez had shown in previews retrospective articles the same results against the use of surgery after irradiation [3,4,18].

In a Radiation Therapy Oncology Group (RTOG 84/20) and Gynecology Oncology Group (GOG) prospective randomized trial comparing radiation therapy followed or not by extra-facial hysterectomy there was a reduction in pelvic recurrence and an increase in progression free survival for patients submitted to surgery after irradiation. Residual disease on cervical specimen was a strong predictor of disease progression and death [6].

In Brazil, a country with a high incidence of cervical cancer, pre-operative treatment is a common approach recommended by gynecologist surgeons. In part, due to the idea that sexual function could be improved with surgery [25].

Our study showed that pelvic radiotherapy followed by high dose rate brachytherapy and hysterectomy yield a 5-year OS of 63% and CSS of 73%. These results are similar to our own experience with exclusive RT and to other published data from other institutions [5,26-28].

Some authors argue that preoperative radiotherapy carry higher rates of toxicity than each modality alone [2,16], but it is definitely not a consensus [2-4,6,18,24]. In the RTOG 84/20 both RT alone and RT→S were well tolerated producing similar rates of grade 3 or 4 adverse effects [6]. It is important to notice that the literature describes higher rates of toxicities in patients submitted to radiotherapy after surgery, and that most of the patients submitted to surgery as a sole treatment in intent, will later need to be irradiated as shown by Landoni and cols [5] who have noticed that up to two thirds of patients submitted to surgery will need adjuvant radiotherapy. In our study RTOG grade 3 and 4 morbidity was rarely seen (genitourinary 9% and gastrointestinal 4%), and were comparable to results of RT alone [6,24]. The incidence of toxicity may also be dependent on total RT dose as the RTOG 84/20 and the current study has used lower brachytherapy doses.

There are a few reasons that may justify the use of post irradiation surgery. They are mostly related to the accomplishment of the pathological staging of the tumor and to the access of *in vivo *response to the previous treatment. Lymph node metastases are known to carry a worse prognosis before treatment [1,29-31]. They also carry a worse prognosis if they remain affected after irradiation [4,6,19]. The evaluation of cervical residual disease also allow the demonstration of tumor sensitivity to radiation and its impact on treatment results [4,6,7,17,18,20,21,32,33]. Also, in the future with the study of genetic and bio-molecular features it may be possible to relate genetic expression with tumor response to radiotherapy.

Our data confirm that the extent of lymph node involvement affects outcome. In the 54 patients submitted to lymphadenectomy, 5-year OS, CSS and DFS were significantly lower in patients who were N+ (p = 0.01, p = 0.01 and 0.005, respectively). Worse 5-years DFS was mainly due to higher distant metastasis rate (p = 0.03) rather than due to local-regional recurrence (p = 0.08) and suggests the need of therapy that could positively impact on distant control. In fact, the standard approach for advanced cervical cancer has been changed after 3 randomized trials and a meta-analysis demonstrate significant benefit of concomitant chemoradiotherapy compared to radiation alone. This Cochrane meta-analysis have found that cisplatin-based chemoradiation improved overall survival, progression free survival and was associated with a significant decrease in local and distant failure compared with radiation alone. The prospective randomized trial GOG #123 compared pre-operative chemoradiotherapy to pre-operative radiotherapy and demonstrated a better outcome for the combined treatment group (for both OS and PFS and also improved the metastasis free survival) [12]. The question to be answered now is whether combined pre-operative chemoradiation is better than combined chemoradiation alone.

The impact of pathological response to radiotherapy on outcome is debatable. Some studies with post-radiation hysterectomy noticed, however, that patients with residual disease on cervical specimens were found to have worse prognosis [6,34]. The biomolecular pathway are being discovered and better-defined. If we might predict which patients would go worse with radiation therapy only, then, we might add hysterectomy. In our data we found 43% CPR on the cervix, but could not demonstrate the relation between cervical CPR and outcome (p = 0.08), possibly because of the small number of studied specimens. Unfortunately, the GOG 123# trial has not analyzed their results on pathological response with chemotherapy.

Maruyama and cols [34] have addressed this subject in their patterns of care study and have found a higher incidence of local and regional recurrence in patients with residual disease on surgical specimens. Thus, they suggested that the addition of more brachytherapy to the vaginal vault could improve results (EBRT→Braqui→Surgery→residual tumor on specimen→vaginal vault brachytherapy). On our study, of the 38 patients who had residual disease on the cervix, 11 were submitted to additional vaginal vault brachytherapy and they did not perform better than the other 27 who were not submitted to extra brachytherapy (p = 0.58).

In our study the time between preoperative RT and surgery higher than 80 days was significantly associated with complete pathological response. As we also showed that CPR was predictive of higher local control it may be important to determine the best interval between RT and S to achieve the best results regarding local control.

Also of great importance is the fact that for exclusive radiotherapy the total time to complete the course of treatment is determinant of outcome as shown by Ferrigno and cols [26]. Considering that patients who receive EBRT and reduced dose HDRB are supposed to undergo surgery the coordination between the radiation oncologist and the surgeon is fundamental. If the patient for any reason is deemed surgery she has to complete the adequate dose of HDRB in the proper length of time.

Of note is the fact that the present study has not used LDRBT, but only HDRB. Lambin and cols [35] studied pathological response following LDRB and found different response rates for small variations in dose rate employed. In a next study we intend to compare pathological response between LDRB and HDRB and relate it to their biological equivalence.

## Conclusion

Time allowed between RT and surgery correlated with pathological response. Pelvic pathological response was associated with improved outcome. Postoperative additional HDRB did not improve therapeutic results. Treatment was well tolerated.
